# Proteolysis of tau by granzyme A in tauopathies generates fragments that are aggregation prone

**DOI:** 10.1042/BCJ20240007

**Published:** 2024-09-18

**Authors:** James P. Quinn, Kate Fisher, Nicola Corbett, Stacey Warwood, David Knight, Katherine A.B. Kellett, Nigel M. Hooper

**Affiliations:** 1 Division of Neuroscience, School of Biological Sciences, Faculty of Biology, Medicine and Health, University of Manchester, Manchester M13 9PT, U.K.; 2 Geoffrey Jefferson Brain Research Centre, Manchester Academic Health Science Centre, Northern Care Alliance & University of Manchester, Manchester, U.K.; 3 Biological Mass Spectrometry Core Research Facility, Faculty of Biology, Medicine and Health, University of Manchester, Manchester M13 9PT, U.K.

**Keywords:** Alzheimers disease, granzyme, protease, tau proteins, tauopathy

## Abstract

Tauopathies, including Alzheimer's disease, corticobasal degeneration and progressive supranuclear palsy, are characterised by the aggregation of tau into insoluble neurofibrillary tangles in the brain. Tau is subject to a range of post-translational modifications, including proteolysis, that can promote its aggregation. Neuroinflammation is a hallmark of tauopathies and evidence is growing for a role of CD8^+^ T cells in disease pathogenesis. CD8^+^ T cells release granzyme proteases but what role these proteases play in neuronal dysfunction is currently lacking. Here, we identified that granzyme A (GzmA) is present in brain tissue and proteolytically cleaves tau. Mass spectrometric analysis of tau fragments produced on digestion of tau with GzmA identified three cleavage sites at R194-S195, R209-S210 and K240-S241. Mutation of the critical Arg or Lys residues at the cleavage sites in tau or chemical inhibition of GzmA blocked the proteolysis of tau by GzmA. Development of a semi-targeted mass spectrometry approach identified peptides in tauopathy brain tissue corresponding to proteolysis by GzmA at R209-S210 and K240-S241 in tau. When expressed in cells the GzmA-cleaved C-terminal fragments of tau were highly phosphorylated and aggregated upon incubation of the cells with tauopathy brain seed. The C-terminal fragment tau_195–441_ was able to transfer between cells and promote aggregation of tau in acceptor cells, indicating the propensity for such tau fragments to propagate between cells. Collectively, these results raise the possibility that GzmA, released from infiltrating cytotoxic CD8^+^ T cells, proteolytically cleaves tau into fragments that may contribute to its pathological properties in tauopathies.

## Introduction

In a number of different dementias, including Alzheimer's disease (AD), corticobasal degeneration (CBD) and progressive supranuclear palsy (PSP), the metabolism of the microtubule-associated protein, tau, is altered [1]. In these so-called tauopathies, the physiological role of tau in promoting microtubule formation and stability is compromised [2–4]. In parallel, through the formation of various soluble and oligomeric species, which ultimately are incorporated into insoluble neurofibrillary tangles, tau exerts toxic effects on neurons [5,6]. In addition, the soluble species of tau can spread through the brain during disease progression affecting neighbouring cells [7,8].

Tau is subject to a range of post-translational modifications, including phosphorylation, acetylation, deamidation, glycosylation, sumoylation, ubiquitylation and proteolysis [3,4,9]. These post-translational modifications can alter the ability of tau to bind and stabilise microtubules and/or facilitate its propensity to aggregate. Although phosphorylation of tau is the most well characterised post-translational modification of the protein, it is becoming apparent that other modifications also critically influence the role of tau in tauopathies [3,4]. In particular, the proteolysis of tau has emerged as a key initiator of neuronal cell death [10–12]. Proteolytic cleavage of tau exacerbates its aggregation, possibly due to disruption of the tertiary structure of tau, and/or by generating fragments that are either directly neurotoxic or propagate transcellularly to promote toxicity in neighbouring cells in the brain [13–16]. A better understanding of the proteases involved in cleaving tau in tauopathies, and of the action of the resulting fragments, will aid understanding of the molecular mechanisms underlying disease pathogenesis and may reveal new targets for therapeutic intervention in tauopathies.

Neuroinflammation is a pathological hallmark of AD and other tauopathies with evidence building for a role of the adaptive immune response in these diseases [17–19], with a growing number of studies implicating peripheral T cells, particularly cytotoxic CD8^+^ T cells, in disease pathogenesis. CD8^+^ T cells were reported to be recruited into the hippocampus of both APP-PS1 mice and AD patients [20–22] and hippocampal T cell infiltration promoted cognitive decline in a mouse model of AD [23]. CD8^+^ T cells were shown to infiltrate the hippocampus and cortex in an age- and pathology-dependent manner in APP-PS1 mice, and increased numbers of CD8^+^ T cells were present in human AD hippocampus where the CD8^+^ T cells were located in the brain parenchyma and positively correlated with Braak staging [24]. In another study, immune cells isolated from the blood of healthy people, and people with mild cognitive impairment (MCI) or AD, revealed a subset of CD8^+^ T cells specifically associated with MCI and AD [25]. In a separate AD cohort, the increased presence of this subset of CD8^+^ T cells in the blood was associated with compromised cognitive performance [25]. In the brains of people who had died with AD, CD8^+^ T cells were present in the perivascular space around the brain's blood vessels and at sites of amyloid-β deposition [25], suggesting recruitment of T cells from the blood to sites of damage in the brain. In people with AD, the CD8^+^ T cell population was enriched in the hippocampus and associated with microtubule associated protein 2 positive neuronal processes [25]. Differential expression analysis of the CD8^+^ T cell subsets that were highly expanded in MCI and AD revealed increased expression of the cytotoxic effector genes, *GZMA*, *GZMH* and *GZMK* that encode the granzyme proteases (three out of the seven genes with highest fold change) [25]. Furthermore, granzyme A (GzmA) protein was localised by immunohistochemistry to CD8^+^ T cells and a higher percentage of GzmA^+^ CD8^+^ cells were detected in hippocampi from AD patients than in individuals without AD [25]. Recently, cytotoxic T cells were found to be markedly increased in areas of the brain with tau pathology in transgenic mice with tauopathy and in individuals with AD, and T cell infiltration was shown to drive tau-mediated neurodegeneration [26], and CD8^+^ T cells induced plaque and tangle-like deposition, modulated AD-related genes and promoted neurodegeneration [27]. However, what is currently lacking is any understanding of the molecular mechanisms by which granzymes released from CD8^+^ T cells might contribute to neurodegeneration.

Granzymes are a family of serine proteases that, together with perforin, are released by cytotoxic lymphocytes into infected or compromised cells. Perforin creates short-lived pores in the plasma membrane of target cells allowing the passage of granzymes into the cytosol where they carry out their effector functions [28]. Granzymes can also be released into the extracellular milieu where they regulate different extracellular processes independently of their ability to induce cell death [29]. There are five granzymes in humans (A, B, H, K and M), with GzmA and granzyme B (GzmB) being the most abundant. Although granzymes induce targeted cell death through non-redundant diverse pathways, also they induce tightly regulated signalling but not indiscriminate protein digestion [30]. Thus, through targeting different proteins, granzymes can exert functions distinct to those directly involved in promoting cell death.

As GzmA and GzmB are the most abundant granzymes, and in the AD brain there was increased expression of GzmA but not GzmB [25], we focused our attention on the potential role of GzmA in the proteolytic cleavage of tau. Here, through studies with recombinant proteins, cellular models and human brain tissues, we show for the first time that GzmA proteolytically cleaves tau at distinct sites to generate N- and C-terminal fragments. The resulting C-terminal fragments are hyperphosphorylated and have the propensity to aggregate and to seed aggregation in neighbouring cells. Using a semi-targeted mass spectrometry (MS) approach we show that peptides corresponding to proteolytic cleavage of tau by GzmA are present in the brain in tauopathies.

## Results

### Granzyme A proteolytically cleaves tau at three distinct peptide bonds

**Figure 1.**
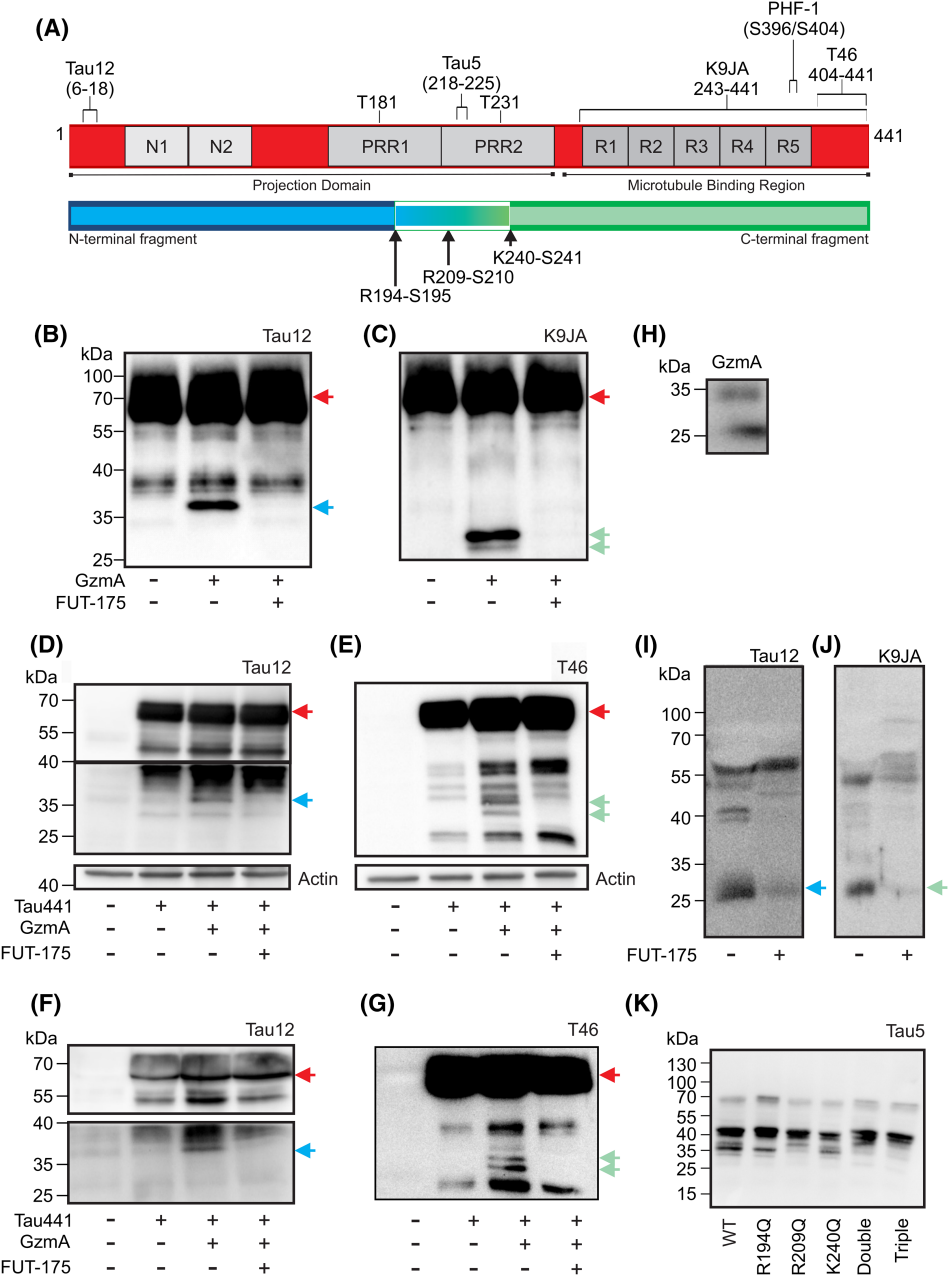
Granzyme A proteolytically cleaves tau. (**A**) Schematic diagram showing the domain organisation of tau and the antibody recognition sites across full-length tau (red), the GzmA cleavage sites in tau and the resultant tau N-terminal fragments (blue) and C-terminal fragments (green). PRR, proline rich region. Recombinant human tau_1-441_ (100 ng) was incubated with activated recombinant GzmA (20 ng) for 1 h at 37°C in the absence or presence of 2 μM FUT-175 and the samples analysed by SDS PAGE and immunoblotted with (**B**) the N-terminal tau antibody Tau12, and (**C**) the C-terminal tau antibody K9JA. HEK293 cells were transfected with the cDNA encoding full-length tau and with the cDNA encoding GzmA and incubated in the absence or presence of 50 μM FUT-175 for 24 h. Cells and conditioned media were harvested, lysates prepared and conditioned media concentrated for analysis. Lysates were immunoblotted with (**D**) the N-terminal tau antibody Tau12 (top) and actin (bottom) and (**E**) the C-terminal tau antibody T46 (top) and actin (bottom). Conditioned media was immunoblotted with (**F**) the N-terminal tau antibody Tau12 and (**G**) the C-terminal tau antibody T46. NK-92 cells were incubated for 48 h in the absence or presence of 50 μM FUT-175 and conditioned media harvested for immunoblot analysis with (**H**) a GzmA antibody, (**I**) the tau antibody Tau12 and (**J**) the tau antibody K9JA. (**K**) HEK293 cells were transfected with the cDNA encoding GzmA along with cDNA encoding full-length tau_1–441_ (WT) or tau constructs containing single point mutations at the identified GzmA cleavage sites (R194Q, R209Q or K240Q), the double point mutation (R194Q, R209Q) or the triple point mutation (R194Q, R209Q, K240Q). Cells were harvested and lysates immunoblotted with the tau antibody Tau5. In panels **D** and **F** the horizontal line indicates that a longer exposure time was used for the lower section. Red arrows indicate the position of full-length tau, blue arrows the position of N-terminal fragments, and green arrows the position of C-terminal fragments.

To determine whether GzmA could proteolytically cleave tau, recombinant human tau was incubated with recombinant GzmA in the absence or presence of the serine protease inhibitor FUT-175 and site-specific antibodies used to identify any fragments produced (Figure 1A). GzmA cleaved tau into a 37 kDa N-terminal fragment and two C-terminal fragments of 30 and 32 kDa (Figure 1B,C). The production of these fragments was completely blocked in the presence of the inhibitor FUT-175 (Figure 1B,C), indicating that they were produced due to the catalytic activity of GzmA. To identify the precise GzmA proteolytic cleavage sites within tau, recombinant human tau was incubated with recombinant GzmA, the fragments separated by SDS PAGE (Supplementary Figure S1), and the tau fragments in the range 30–37 kDa digested with AspN and then analysed by LC–MS/MS (Supplementary Figure S2). This analysis of the tau fragments produced by GzmA identified three distinct proteolytic cleavage sites within tau at R194-S195, R209-S210 and K240-S241 (Figure 1A), consistent with the specificity of GzmA to cleave after Arg and Lys (https://www.ebi.ac.uk/merops/cgi-bin/pepsum?id=S01.135;type=P).

Co-expression of full-length human tau with GzmA in HEK cells resulted in the appearance of N- and C-terminal fragments of tau in both the lysates (Figure 1D,E) and conditioned media (Figure 1F,G) that were similar in size to those observed in the recombinant protein assay (Figure 1B,C). The pattern of C-terminal fragments observed with the C-terminal antibody (T46) in Figure 1E and F was identical with that observed with the C-terminal K9JA antibody (Supplementary Figure S3), consistent with the overlapping epitopes recognised by these two antibodies (Figure 1A). The production of the N- and C-terminal fragments was blocked in the presence of the inhibitor FUT-175 (Figure 1D–G; Supplementary Figure S3). Immunoblotting of conditioned media from natural killer NK92 cells, which release cytotoxic granules containing GzmA (Figure 1H) and also endogenously express tau, indicated the presence of similar N- and C-terminal GzmA-cleaved tau fragments whose production was reduced by the serine protease inhibitor FUT-175 (Figure 1I,J).

To further investigate the proteolytic cleavage of tau at the R194-S195, R209-S210 and K240-S241 peptide bonds by GzmA, site-directed mutagenesis was performed to replace R194, R209 and K240 with glutamine which is non-permissive for GzmA cleavage (https://www.ebi.ac.uk/merops/cgi-bin/pepsum?id=S01.135;type=P). Single, double and the triple point mutant constructs were expressed alongside GzmA in HEK cells and the tau fragment profile assessed by immunoblotting. In cells expressing wild-type tau, fragments of 37, 35 and 33 kDa were detected with the tau antibody Tau5 (Figure 1K). In cells expressing the R194Q mutant of tau, the 35 and 33 kDa fragments were still present, but not the 37 kDa fragment. While in the cells expressing the R209Q fragment the 37 kDa and, to a lesser extent, the 35 kDa fragments were visible, but not the 33 kDa fragment. In cells expressing the K240Q mutant all three fragments were still detected. In cells expressing either the double or triple point mutants, all three fragments were reduced. These results suggest that the 37 kDa fragment is a C-terminal fragment predominantly generated by GzmA cleavage of tau at the R194-S195 peptide bond and the 33 kDa fragment is a C-terminal fragment predominantly generated by proteolytic cleavage at the R209-S210 peptide bond. Under the conditions employed in this experiment, proteolytic cleavage of tau by GzmA at the K240-S241 peptide bond appeared to be minimal. Together these data indicate that GzmA proteolytically cleaves tau at multiple sites in both recombinant and cell-based assays.

### Tau fragments corresponding to proteolysis by granzyme A are present in tauopathy brain

**Figure 2.**
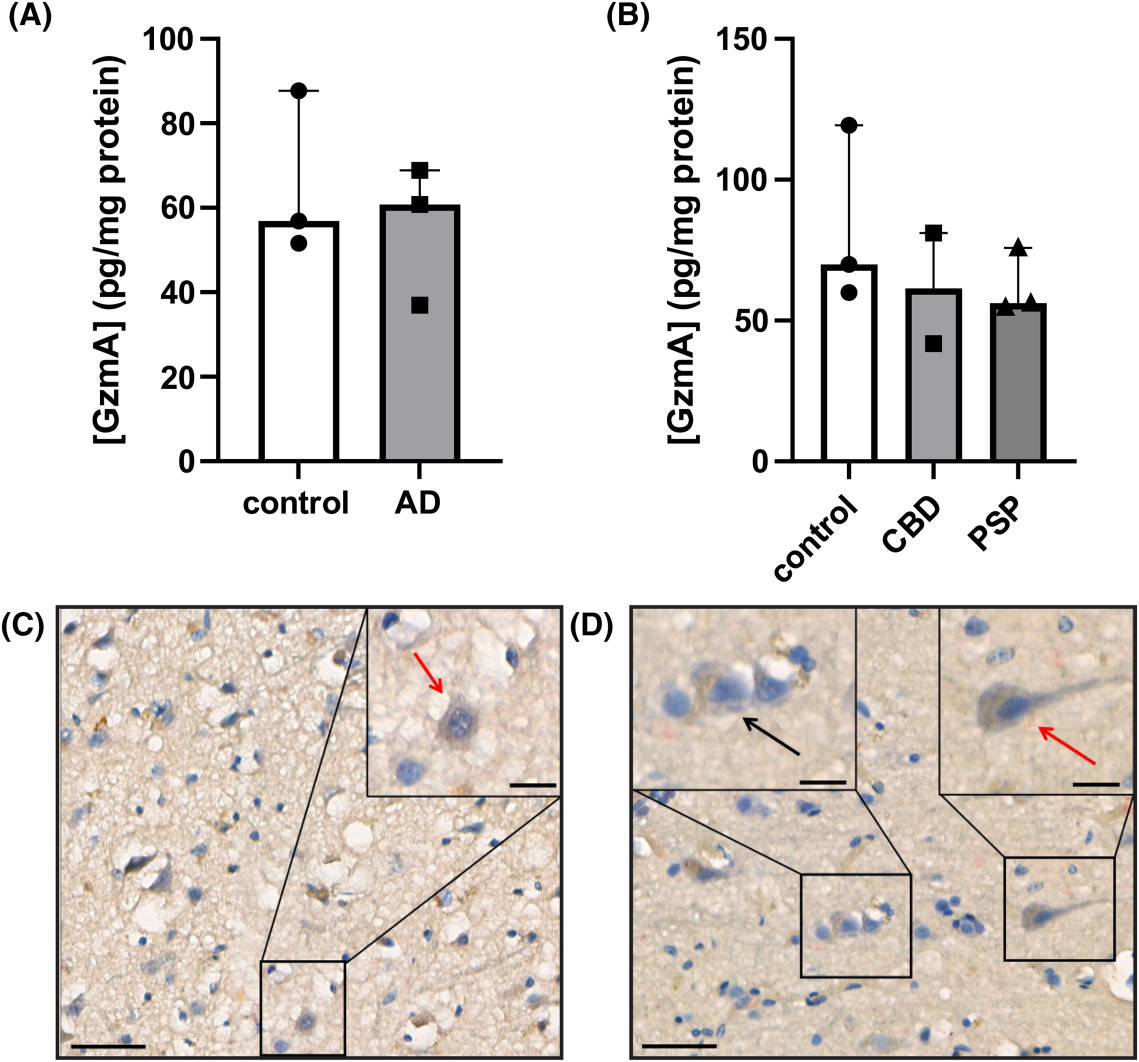
Granzyme A is present in human tauopathy brain. (**A**) GzmA levels in AD and control brain samples from temporal cortex as determined by ELISA. Median with range, *n* = 3 per group. (**B**) GzmA levels in CBD, PSP and control brain samples from pre-motor cortex as determined by ELISA. Median with range, *n* = 3 for control and PSP, *n* = 2 for CBD. GzmA expression was analysed by immunohistochemistry within neurons from the pre-motor cortex of PSP case 11/10 (**C**) and control case 14/08 (**D**) (*n* = 3; three sections per brain). To determine whether GzmA was expressed within neurons, an antibody against GzmA was used in conjunction with the refine red chromogen, along with an antibody against the neuronal marker, NeuN, used in conjunction with the brown chromogen, 3, 3′-diaminobenzidine, and H&E, a blue nuclear marker. Scale bar = 40 μm. Red arrow highlights magnified GzmA-positive neurons and black arrow highlights a magnified GzmA-negative neuron. Scale bar = 15 μm for magnified images.

To be relevant to disease pathogenesis both GzmA protein and its tau cleavage products should be present in tauopathy brain tissue. First, using an ELISA, GzmA was detected in the temporal cortex of AD brains (Figure 2A) and in the pre-motor cortex of CBD and PSP brains (Figure 2B), as well as in age-matched control brains. Second, we used immunohistochemistry to assess the presence of GzmA in a PSP brain (Figure 2C) and a control brain (Figure 2D), with the GzmA showing intraneuronal staining in both cases. These data suggest that GzmA is present in both tauopathy and control brain samples (discussed later). Then, to determine whether tau fragments corresponding to proteolytic cleavage by GzmA were present in tauopathy brain, we developed a semi-targeted mass spectrometry methodology that uses synthetic peptides based on the proposed GzmA cleavage sites in tau to direct analysis in a highly sensitive and specific manner. Tau and its fragments were first immunoprecipitated from the temporal cortex from an AD and a PSP brain. Following separation by SDS PAGE, the protein bands were subjected to protease digestion with either Asp-N or Lys-C and the digested samples analysed by nano-liquid chromatography-mass spectrometry. Peptides corresponding to proteolytic cleavage of tau at R209-S210 and K240-S241 were positively identified in both the AD and PSP brains (Supplementary Figures S4 and S5). These data indicate that fragments of tau corresponding to proteolysis by GzmA are present in tauopathy brain.

### Granzyme A-cleaved C-terminal tau fragments are highly phosphorylated

**Figure 3.**
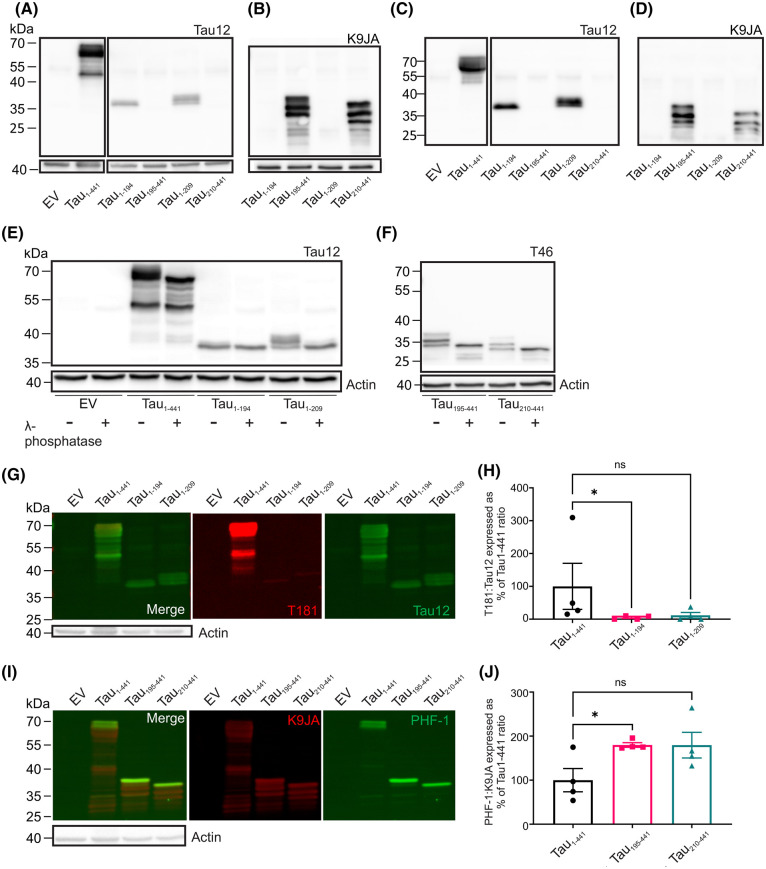
Granzyme A cleaved C-terminal, not N-terminal, tau fragments are highly phosphorylated compared with full-length tau. HEK293 cells expressing either an empty vector (EV), full-length tau (Tau_1–441_) or with constructs to express the N- and C-terminal fragments that arise from GzmA cleavage of tau at the R194-S195 (tau_1–194_, tau_195–441_) and R209-S210 (tau_1–209_, tau_210–441_) peptide bonds. Cells and conditioned media were harvested. Lysates were blotted with (**A**) tau antibody Tau12 (top panel) and actin (bottom panel) and (**B**) tau antibody K9JA (top) and actin (bottom). Conditioned media samples were blotted with (**C**) tau antibody Tau12 and (**D**) tau antibody K9JA. Following expression of the constructs in HEK293 cells, the lysates were subjected to dephosphorylation using Lambda phosphatase, before being blotted with (**E**) the tau antibody Tau12 (top) and actin (bottom) and (**F**) the tau antibody T46 (top) and actin (bottom). Fluorescent antibodies were used to allow comparison of the ratio of p-tau:total tau following transfection of cDNAs encoding full-length tau (tau_1–441_) or the N-terminal fragments (tau_1–194_ and tau_1–209_) immunoblotted with (**G**) the p-tau antibody T181 and the tau antibody Tau12, quantified in (**H**) or with the cDNAs encoding full-length tau (tau_1–441_) or the C-terminal fragments (tau_195–441_ and tau_210–441_) immunoblotted with (**I**) the p-tau antibody PHF-1 and the tau antibody K9JA, quantified in (**J**). Lower panels in (**G**) and (**I**) show actin. Kruskal–Wallis test with Dunn's multiple comparisons test (non-parametric as data not normally distributed). Graphs show mean ± SEM, *n* = 4, *P < 0.05, ns, not significant.

Proteolytic cleavage of tau by GzmA at the K240-S241 peptide bond generates a C-terminal fragment that is very similar to those generated upon calpain cleavage of tau at R242-L243 and R230-T231 [11,12]. Such C-terminal fragments have been extensively studied in terms of their phosphorylation status and ability to aggregate [16,31]. Thus, we focused our attention on the larger C-terminal fragments of tau resulting from GzmA proteolysis at R194-S195 and R209-S210 for which there are little data available in respect of their molecular and cellular properties. Hyperphosphorylation of tau is a key component of tauopathy pathogenesis [32] and N-terminally truncated fragments of tau are more highly phosphorylated compared with the full-length protein [31]. To investigate the phosphorylation status of the GzmA-cleaved N- and C-terminal fragments of tau, the cDNAs encoding the GzmA-cleaved tau fragments tau_1–194_, tau_195–441_, tau_1–209_ and tau_210–441_ (corresponding to proteolytic cleavage of tau by GzmA at the R194-S195 and R209-S210 peptide bonds) were transfected into HEK cells. All four tau fragments were expressed and present in both cell lysates and conditioned media, indicating that the fragments are expressed by the cells and secreted into the medium (Figure 3A–D). The C-terminal fragments, in particular, appeared as multiple bands on the immunoblots which may be as a result of phosphorylation. Indeed, dephosphorylation with lambda-phosphatase revealed that the tau_1–209_, tau_195–441_ and tau_210–441_ fragments were variably phosphorylated (Figure 3E,F). Further analysis with the phosphorylation site-specific antibodies T181 and PHF-1 (S396/S404) revealed that the C-terminal, but not the N-terminal, GzmA-cleaved tau fragments were more highly phosphorylated than full-length tau (Figure 3G–J).

### Granzyme A-cleaved tau fragments have increased aggregation and seeding propensity

**Figure 4.**
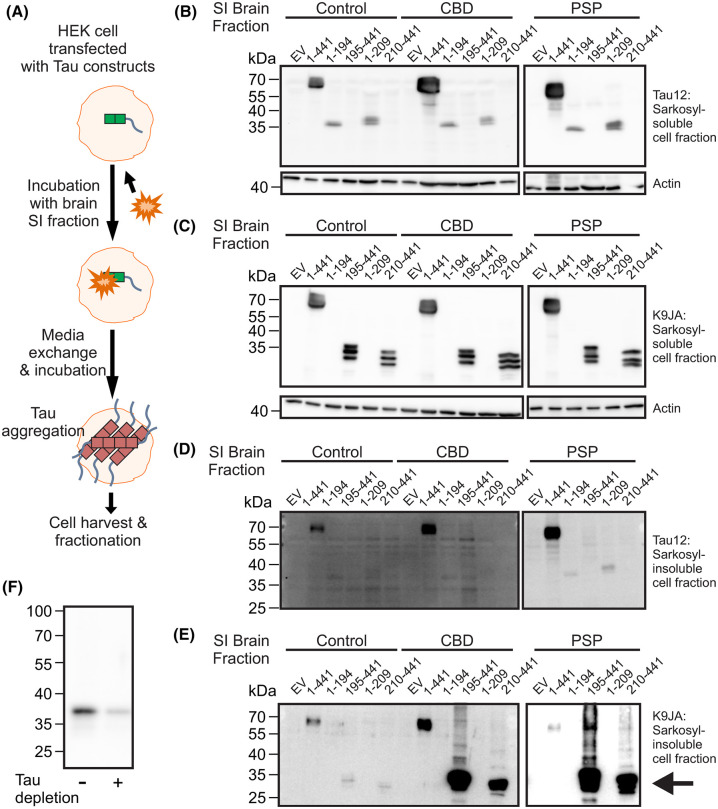
Granzyme A-cleaved tau fragments have an altered aggregation propensity. (**A**) Schematic outlining the experimental protocol. HEK cells expressing an empty vector (EV), full-length tau (tau_1–441_) or tau fragments (1–194, 195–441, 1–209 and 210–441) were incubated for 6 h with brain SI fraction extracted from either control, CBD or PSP brains. Following media exchange to remove the brain SI fraction, the cells were incubated for 48 h to allow tau to aggregate. Cells were then harvested in homogenisation buffer (10 mM Tris–HCl, 4 M NaCl, 1 mM EGTA, 1 mM DTT, pH 7.5) and subjected to Sarkosyl fractionation to generate the soluble and insoluble fractions. The resulting Sarkosyl-soluble fraction was immunoblotted with (**B**) the tau antibody Tau12 (top panel) and actin (bottom panel) and (**C**) the tau antibody K9JA (top) and actin (bottom). The resulting SI fraction was immunoblotted with (**D**) the tau antibody Tau12 and (**E**) the tau antibody K9JA. (**F**) Tau was immunodepleted from the CBD SI sample using a combination of tau antibodies (HT7, Tau5, PHF-1) before incubation with HEK cells expressing the 195–441 fragment. Following incubation, cells were harvested, fractionated and the resulting SI sample immunoblotted with the tau antibody K9JA. In (**E**) the arrow indicates the position of tau fragments that are prone to aggregation.

Fragments of tau have an increased ability to aggregate and propagate aggregation between cells compared with full-length tau, indicating that tau truncation may play a critical role in tau pathogenesis [33,34]. To explore the aggregation and seeding potential of the GzmA-cleaved tau fragments, an established protocol [16,35] using as ‘seed’ the SI fraction from CBD or PSP brain (in which aggregated C-terminal tau fragments are abundant [36,37]) was employed (Figure 4A). HEK cells expressing either full-length tau_1–441_ or one of the GzmA-derived tau fragments (tau_1–194_, tau_195–441_, tau_1–209_, tau_210–441_) were incubated with SI fractions from either control, CBD or PSP brain. The medium was then exchanged and the cells incubated for a further period. The cells were then harvested, fractionated into the sarkosyl-soluble and SI fractions and these fractions immunoblotted with either the N-terminal antibody Tau12 or the C-terminal antibody K9JA (Figure 4B–E). In the cells expressing full-length tau_1–441_, incubation with either CBD or PSP brain seed resulted in an increase in tau aggregates in the SI fraction compared with cells incubated with the control brain seed (Figure 4D,E). In cells expressing the N-terminal fragments tau_1–194_ and tau_1–209_, minimal aggregation of the fragments occurred as seen by the lack of the fragment in the SI fractions following incubation with the CBD or PSP brain seeds (Figure 4D,E), although the fragments were clearly detected in the sarkosyl-soluble fractions (Figure 4B,C). In cells expressing the C-terminal fragments tau_195–441_ and tau_210–441_ there was increased aggregation of truncated tau when incubated with the CBD and PSP brain seeds compared with cells expressing full-length tau but no aggregation was seen with the control brain seed (Figure 4D,E). Immunodepletion of tau from the CBD brain seed reduced the amount of aggregation seen with the tau_195–441_ fragment (Figure 4F), indicating that the aggregation is dependent on tau in the original brain sample.

**Figure 5.**
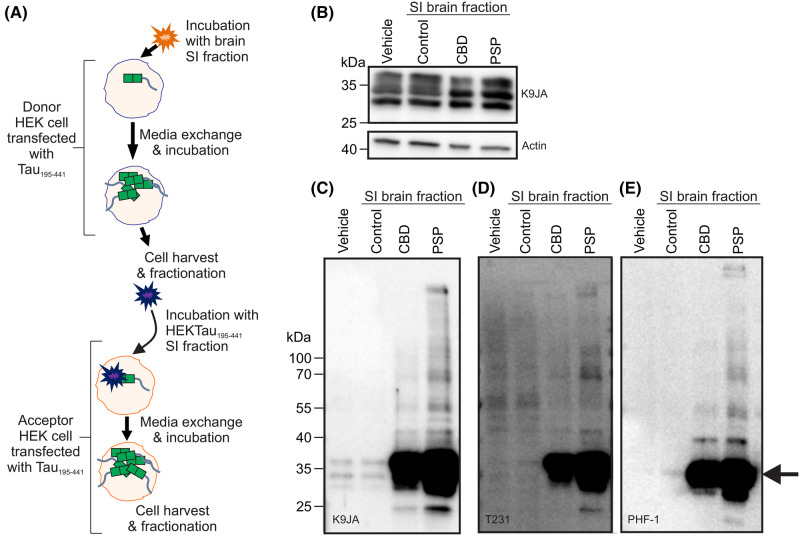
The granzyme A-cleaved fragment tau_195-441_ has increased seeding propensity. (**A**) Schematic outlining the experimental protocol. HEK cells expressing tau_195–441_ were incubated with vehicle or SI fractions from control, CBD or PSP brain samples to promote aggregation of the tau_195–441_ fragment in the cell. These cells, the ‘donor cells’ were then harvested and an SI fraction prepared for subsequent incubation with cells (‘acceptor cells’) expressing tau_195–441_. Aggregation in the SI fraction of the acceptor cells was then assessed. (**B**) The soluble fraction from the acceptor cells was immunoblotted with the tau antibody K9JA. The SI fraction from the acceptor cells was immunoblotted with tau antibodies K9JA (**C**), PHF-1 (**D**) and T231 (**E**). The arrow indicates the position of tau fragments that are prone to aggregation.

To determine whether the GzmA-cleaved tau fragments could propagate aggregation between cells, HEK cells expressing the most aggregation prone fragment, tau_195–441_, were incubated with vehicle or the SI fraction from control, CBD or PSP brain samples to promote aggregation of the tau_195–441_ fragment in the cell (Figure 5A). These cells, the ‘donor cells’ were then harvested and an SI fraction prepared. ‘Acceptor cells’ expressing tau_195–441_ were then incubated with the SI fraction from the donor cells and aggregation in the acceptor cells assessed (Figure 5A). The detection of tau_195–441_ in the sarkosyl-soluble fraction from the acceptor cells was unchanged regardless of the brain sample used for the initial seeding (Figure 5B). In the SI fraction from the acceptor cells, however, the initial seeding with CBD or PSP brain samples caused increased tau aggregation compared with the control brain sample or vehicle (Figure 5C). The resulting aggregated tau_195–441_ was phosphorylated as shown by its recognition with the phospho-epitope antibodies Thr231 and PHF-1 (Figure 5D,E). Together, these data indicate that the GzmA-cleaved C-terminal tau fragments, but not the N-terminal tau fragments, have a high capacity to aggregate in cells and can efficiently transfer aggregated conformations between cells.

## Discussion

The proteolytic fragmentation of tau plays a crucial role in the pathogenesis of many different tauopathies (reviewed in [11,12]). A number of different proteases have been identified to cleave tau, including calpains, caspases, cathepsins and asparagine endopeptidase (reviewed in [11,12]). Here, we identified the serine protease GzmA as capable of cleaving tau at, at least, three distinct peptide bonds: R194-S195, R209-S210 and K240-S241. *GZMA* transcripts have been detected in tauopathy brains and GzmA was localised by immunohistochemistry to CD8^+^ T cells in the hippocampus of an AD-affected brain [25]. Here, using an ELISA and immunohistochemistry, we detected GzmA protein in the brains from individuals with AD, CBD and PSP, as well as control brains. Using a semi-targeted mass spectrometry approach we were able to detect peptides corresponding to proteolytic cleavage of tau by GzmA in AD and PSP brains, suggesting that proteolytic cleavage of tau by GzmA may be relevant to disease pathogenesis.

Granzymes, together with perforin, are the main components of the cytotoxic lymphocyte granule exocytosis pathway. This pathway is a specialised form of intracellular protein delivery by which lymphocytes release granzymes and perforin into target cells. Perforin creates short-lived pores in the plasma membrane of the target cells allowing the granzymes to pass into the cytosol [28]. Most of our data, including the coexpression of tau and GzmA in HEK cells, along with immunohistochemistry showing that GzmA is present inside neurons in the human brain, would be consistent with GzmA proteolytically cleaving tau intracellularly. This scenario that GzmA acts within neurons is consistent with a recent study showing that perforin was required for CD8^+^ T cells to initiate AD-like pathophysiology [27]. In light of these observations, it would be interesting to assess the presence of perforin in tauopathy brain, although the short-lived perforin pores may not be present at end stage disease. Granzymes can also be released into the extracellular milieu where they regulate different extracellular processes independently of their ability to induce cell death through entering the cell via perforin pores [29]. There is also the possibility that GzmA acts on full-length or even partially truncated tau that has been secreted into the extracellular milieu [38]. This scenario would be consistent with data showing expression of GzmA adjacent to MAP2^+^ neuronal processes in AD brain [25].

To our knowledge, this is the first time that GzmA protein levels have been measured in tauopathy and control brain samples. Interestingly, there was no significant difference in the amount of GzmA detected by ELISA in the tauopathy versus the control brains. This may reflect the relatively small sample size (*n* = 3), that even in aged control brains the amount of GzmA is relatively high, or indeed that GzmA is not the most relevant granzyme targeting tau in tauopathies (discussed further below). Although intraneuronal GzmA staining was observed in both a PSP and a control brain, a more extensive analysis is required to determine the precise cellular and subcellular localisation of GzmA in the brain and whether there is greater colocalization of GzmA with tau in tauopathy compared with control brains.

Our approach using recombinant tau and GzmA in enzymatic assays, cellular expression of GzmA and mutation of key amino acids at the predicted GzmA cleavage sites in tau provide independent validation that the tau fragments observed are due to the action of GzmA and not another granzyme or unrelated protease. The general serine protease inhibitor FUT-175 was used to block GzmA activity as there is no specific small molecule inhibitor of GzmA. Genetic knockdown of GzmA in animal models of tauopathies could be used in future studies to confirm a role for the protease in the proteolytic cleavage of tau and the impact this has on the development of neurodegeneration.

There are numerous proteolytic fragments of tau that have been detected in the brain for which the responsible protease has yet to be identified [12,39] and, based on the identified cleavage sites within tau, some of these fragments could potentially be generated by the action of GzmA. For example, GzmA cleavage of tau at the R194-S195 peptide bond could potentially produce the C-terminal tau35 fragment whose N-terminal residue was determined as S195 [36]. When expressed in cells, tau35 compromised microtubules, disrupted insulin signaling and induced the unfolded protein response [40]. Transgenic mice expressing tau35 recapitulated key features of human tauopathies, including aggregated and abnormally phosphorylated tau, progressive motor and cognitive deficits, autophagic and lysosomal dysfunction, loss of synaptic protein and reduced life-span [41]. Proteolytic cleavage of tau at the K240-S241 peptide bond by GzmA followed by aminopeptidase activity could contribute to the formation of tau-CTF24, a 24 kDa C-terminal fragment of tau that was identified in the brains of Tg601 tauopathy mice and whose N-terminus was identified by mass spectrometry as L243 [16]. Proteolysis of tau by GzmA at R209-S210 and K240-S241 could contribute to the formation of the two N-terminal fragments of tau (NT1 and NT2), respectively [42]. Based on antibody epitopes, NT1 had a minimal sequence of 6–198, while NT2 had the larger sequence 6–224 [42]. Both NT1 and NT2 were elevated in the CSF and plasma of AD individuals with NT1 levels separating controls from those with AD [42]. In a cohort of clinically normal elderly, plasma NT1 levels at study entry were highly predictive of future cognitive decline, pathological tau accumulation, neurodegeneration, and a transition to a diagnosis of AD [43]. In the cerebrospinal fluid of AD individuals, C-terminal fragments of tau containing the residues 243–254, which are present in all the GzmA-cleaved C-terminal fragments, were the most highly correlated with tau positron-emission tomography and cognitive measures [44].

The C-terminal GzmA-cleaved tau fragments were more highly phosphorylated than the N-terminal GzmA-cleaved tau fragments, consistent with other reports that C-terminal but not N-terminal fragments of tau are highly phosphorylated [31,40] and that the tau35 fragment and fragments between 30 and 40 kDa were heavily phosphorylated in the brains of tauopathy patients [36,45]. Although the N-terminal fragments were less phosphorylated compared with full-length tau_1–441_, tau_1–209_ was more phosphorylated than tau_1–194_. likely due to seven additional phosphorylation sites between residues 195–209 (S195, Y197, T198, S199, S202, T205 and S208) within the proline-rich region of tau [46]. The GzmA-cleaved C-terminal fragments of tau, tau_195–441_ and tau_210–441_, displayed increased aggregation when incubated with tauopathy brain-derived SI fractions as compared with full-length tau_1–441_. This increased propensity of the C-terminal fragments of tau to aggregate is likely due to a combination of increased phosphorylation compared with tau_1–441_ and exposure of the aggregation-prone regions _275_VQIINK_280_ and _306_VQIVYK_311_ within the microtubule-binding repeat domains [47,48]. Neither of the N-terminal fragments of tau, tau_1–194_ and tau_1–209_, aggregated to the same extent as full-length tau_1–441_, consistent with these fragments lacking the aggregation-prone regions of tau and agrees with previous data showing that the N-terminal tau fragment, tau_1–196_, had a lower propensity to aggregate compared with tau_1–441_ [49].

In tauopathies tau or its fragments act in a ‘prion-like’ mechanism, spreading between cells and seeding aggregation of tau in neighbouring cells (reviewed by [8,50]). The ability of the GzmA-cleaved tau fragment, tau_195–441_, to propagate between cells and seed aggregation in neighbouring cells was examined using cells expressing the fragment incubated in the presence of tauopathy brain material. Only donor cells incubated with tauopathy brain material were able to seed tau aggregation in the acceptor cells, indicating that aggregated tau_195–441_ is able to cause templated seeding in other cells. These results are consistent with those obtained in a similar cell-based assay using tau-CTF24 (tau_243–441_) which also was shown to propagate between cells and seed aggregation in neighbouring cells [16]. The phosphorylation, aggregation and seeding data indicate that GzmA-cleaved C-terminal tau fragments have an increased likelihood to aggregate, to propagate tau aggregation and transcellularly spread, and thus that proteolysis of tau by GzmA could facilitate pathogenesis in tauopathies.

Chronic inflammation, encompassing neuroinflammation and chronic systemic inflammation, have been proposed as a unifying theme underlying multiple tauopathies, with chronic inflammation driving disease progression and influencing disease development [51]. This is reinforced by the observation that in tauopathy (TE4) mice, that display tau aggregation with neurodegeneration, CD8^+^ T cell infiltration drove the neurodegeneration [26]. The CD8^+^ T cell subsets that were highly expanded in MCI and AD had increased expression of *GZMH* and *GZMK*, as well as *GZMA* [25]. More recently, infiltrating CD8^+^ T cells were shown to exacerbate AD pathology in a 3D human neuroimmune model in which higher expression of *GZMA*, *GZMB* and *GZMH* was associated with the cytotoxic CD8^+^ T cells [52]. In preliminary experiments we have evidence that GzmB and granzyme K but not granzyme H can cleave tau into fragments that are of different mass to those produced by GzmA (A. Snedden, K.A.B. Kellett and N.M. Hooper, unpublished). Further work is required to ascertain whether granzymes in addition to, or instead of, GzmA are involved in cleaving tau to generate neurotoxic fragments that play a role in the neurodegenerative process in tauopathies.

In summary, our data demonstrate that GzmA proteolytically cleaves tau into discrete N- and C-terminal fragments, and that these cleavage events are blocked upon either inhibition of GzmA or by mutation of critical amino acid residues at the specific cleavage sites in tau. The resulting GzmA-produced C-terminal, but not the N-terminal, fragments are highly phosphorylated, have an increased propensity to aggregate compared with full-length tau, and can propagate tau aggregation between cells. Mass spectrometric analyses indicates that tau fragments with proteolytic cleavage sites corresponding to the action of GzmA are present in the tauopathy brain. Together these observations suggest that GzmA-cleaved tau fragments may be relevant to disease pathogenesis. Further work is required to provide a direct mechanistic link between infiltrating CD8^+^ T cells in the tauopathy brain, the released granzymes, granzyme-cleaved tau fragments and the downstream neurodegeneration. Elucidating these links may reveal novel therapeutic interventions in tauopathies.

## Materials and methods

### Human brain samples

Tissue samples were supplied by the Manchester Brain Bank, University of Manchester, which is part of the Brains for Dementia Research programme, jointly funded by Alzheimer's Research UK and Alzheimer's Society. Tissue samples were covered under ethics approved by the Newcastle & North Tyneside 1 Research Ethics Committee (09/H0906/52 + 5 and 19/NE/0242) on 6 May 2014 and 25 October 2019. Frozen sections of pre-motor cortex or temporal cortex were obtained from the brains of control, CBD, PSP or AD individuals (Table 1).

**Table 1.** Human brain samples used in the study

Case no.	Pathological diagnosis	Gender	Age at death
14/08	Normal for age	M	85
16/29	Normal for age	M	69
17/38	Normal for age	F	101
18/03	Normal for age	M	88
18/05	Normal for age	M	91
18/39	AD	F	75
19/13	AD	M	74
19/04	AD	M	82
10/32	CBD	M	70
14/33	CBD	M	82
14/40	CBD	M	90
11/10	PSP	M	90
12/31	PSP	F	71
17/33	PSP	M	76
19/05	PSP	F	95
13/06	PSP	M	80

### Cell lines

Human embryonic kidney (HEK) cells containing the T antigen were cultured in Dulbecco's Modified Eagle's Medium (DMEM; Lonza) supplemented with 10% (v/v) foetal bovine serum. NK-92 cells kindly provided by Professor Daniel Davis (University of Manchester, U.K.) were cultured in RPMI-1640 containing 20% (v/v) foetal bovine serum, l-glutamine (200 mM), penicillin (100 IU/ml) and streptomycin (100 μg/ml), 1 M HEPES buffer and interleukin-2 (125 U/ml). All cells were incubated at 37°C in a 5% CO_2_ environment. Cells were regularly checked for mycoplasma infection using the EZ-PCR kit (Biological Industries). For inhibition of GzmA, NK-92 and HEK cells were treated for 48 h with 50 μM FUT-175 (Alfa Aesar) diluted in Opti-MEM (ThermoFisher Scientific).

### Cloning of tau constructs and GzmA

C-terminal tau fragments, tau_195–441_ and tau_210–441_ were subcloned from tau_1-441_ in the mammalian vector pcDNA3.1 (-), kindly provided by Diane Hanger (Kings College London, U.K.) using polymerase chain reaction (PCR) and inserted into pcDNA3.1 (-) using the BamH1 and EcoR1 restriction sites. PCR was performed using Phusion high-fidelity DNA polymerase according to the manufacturer's instructions (New England Biolabs). The PCR product was confirmed by electrophoresis on a 1% (w/v) agarose gel containing SYBR safe green (ThermoFisher Scientific) then cleaned up using the QIAquick PCR purification kit according to the manufacturer's instructions (Qiagen). The purified PCR product was digested using BamH1 and EcoR1 restriction enzymes for 2 h at 37°C. The digestion reaction was stopped by the addition of DNA gel loading buffer (50% (v/v) glycerol, 50% (v/v) ddH_2_O and 0.02% (w/v) bromophenol blue) to the purified PCR products which were assessed using electrophoresis on a 1% (w/v) agarose gel containing SYBR safe green. Digested inserts were excised under uv light and purified using QIAquick gel extraction kit according to the manufacturer's instructions (Qiagen).

Site-directed mutagenesis was used to introduce a glutamine at R194, R209 or K240 in tau_1–441_ using the QuikChange II XL Site-directed Mutagenesis Kit according to the manufacturer's instructions (Agilent Technologies) (see Table 2 for details of the primers used). The resulting constructs encoding single (R194Q, R209Q or K240Q), double (R194Q, R209Q) or triple (R194Q, R209Q, K240Q) point mutations were sequenced to confirm the mutations. N-terminal tau fragments, tau_1–194_ and tau_1–209_, were also generated by site-directed mutagenesis of tau_1–441_ to introduce stop codons at either S195 or S210 using the same kit (see Table 2 for details of the primers used). The cDNA encoding Flag-tagged GzmA was subcloned into the piREShyg vector by GenScript using the Nsi1 and Not1 restriction sites. Successful subcloning was assessed by digesting the vector with Nsi1 and Not1 restriction enzymes for 2 h at 37°C and assessing the product using a 1% (w/v) agarose gel, and the construct confirmed by DNA sequencing. Plasmid DNA was transformed into XL10-Gold Ultracompetent *Escherichia coli* cells according to the manufacturer's instructions (Agilent Technologies) and purified using a Midi-preparation kit following the manufacturer's protocol (Qiagen). Purified plasmid DNA was then sequenced. Samples were stored at −20°C until required for transfections.

**Table 2.** Site-directed mutagenesis primers

Construct	Sequence (5′ > 3′)
Tau_1–441_ R194Q	Forward: CCAAAATCAGGGGATCAGAGCGGCTACAGCAGCC
	Reverse: GGCTGCTGTAGCCGCTCTGATCCCCTGATTTTGG
Tau_1–441_ R209Q	Forward: CTCCCGGCAGCCAGTCCCGCACCCCG
	Reverse: CGGGGTGCGGGACTGGCTGCCGGGAG
Tau_1–441_ K240Q	Forward: GTCTTCCGCCCAGAGCCGCCT
	Reverse: GGCGACTTGGGTGGAGTAC
Tau_1–194_	Forward: AATCAGGGGATCGCTGAGGCTACAGCAGCCC
	Reverse: GGGCTGCTGTAGCCTCAGCGATCCCCTGATT
Tau_195–441_	Forward: GTCTGGATCCCCACCATGAGCGGCTACAGCAGCCCCGGCTCCCCAGGC
	Reverse: TCATGAATTCTCACAAACCCTGCTTGGCCAGGGAGGCAGACACCTCGTC
Tau_1–209_	Forward: ACTCCCGGCAGCCGCTGACGCACCCCG
	Reverse: CGGGGTGCGTCAGCGGCTGCCGGGAGT
Tau_210–441_	Forward: GTCTGGATCCCCACCATGTCCCGCACCCCGTCCCTTCCAACCCCACCCACCCGG
	Reverse: TCATGAATTCTCACAAACCCTGCTTGGCCAGGGAGGCAGACACCTCGTC

### Transfection of cells

HEK cells were transiently transfected with the appropriate cDNA cloned into the pCDNA 3.1 (-) (tau constructs) or pIREShyg (GzmA) vector per well of a six well plate (Corning) using FuGENE HD according to the manufacturer's instructions (Promega). Briefly, cells were plated out 24 h prior to transfection in order to reach 60–70% confluency. GzmA plasmid cDNA (0.5 μg) and tau_1–441_ plasmid cDNA (0.5 μg) were co-transfected per well of a six well plate, and transfections of the different tau constructs were performed using 0.5 μg plasmid cDNA per well of a six well plate. For each reaction, plasmid cDNA was diluted in 200 μl Opti-MEM and 3 μl FUGENE HD (transfection reagent: DNA ratio of 6:1 or 3:1). The transfection complex was briefly vortexed and incubated for 10–15 min at room temperature. Cells were washed once with phosphate-buffered saline (PBS) without metals and fresh Opti-MEM added, and then the entire transfection complex was added dropwise to the cells; no cell culture media changes were performed after transfection.

### Cell lysate and media preparation

For HEK cells, medium was removed and cells washed once on ice with PBS containing metals. Cells were scraped in lysis buffer (50 mM Tris, 150 mM NaCl, 0.5% [w/v] sodium deoxycholate, 1% [v/v] Nonidet-P40, pH 8.0) containing 4% (v/v) protease inhibitor cocktail (EDTA-free PIC; Roche Diagnostics) and 10% (v/v) phosphatase inhibitor (PhosSTOP; Sigma–Aldrich). Cells were resuspended, vortexed and incubated on ice for 30 min, and lysed cells clarified by centrifugation at 14 000 × ***g*** for 10 min at 4°C, transferred to a sterile tube and stored at −20°C. The same was also performed for NK-92 cells, however, the suspension cells were initially centrifuged at 1980 × ***g*** for 5 min at 4°C. Media was removed from the cells and centrifuged at 1980 × ***g*** for 5 min at 4°C to remove cell debris. The supernatant was transferred into a Vivaspin 6 ml column (Generon) with a molecular mass cut off value of 10 kDa. The media was concentrated ∼25 times by centrifugation at 2690 × ***g*** for 45 min at 4°C.

### Sodium dodecyl sulfate polyacrylamide gel electrophoresis and immunoblotting

Lysate and media samples were made up to an equal protein concentration using the bicinchoninic acid (BCA) assay (ThermoFisher Scientific) by diluting in lysis buffer or dH_2_O, respectively. Sodium dodecyl sulfate (SDS) polyacrylamide gel electrophoresis (PAGE) sample buffer (100 mM Tris–HCl, 10% (v/v) glycerol, 2% (w/v) SDS, 0.02% (w/v) bromophenol blue, pH 6.8) was then added and the samples heated at 95°C for 5 min. Proteins were separated by gel electrophoresis using 1.5 mm thick 12–20% acrylamide gradient resolving Tris-glycine gel and a 3% acrylamide stacking Tris-glycine gel with a pre-stained molecular mass ladder (10–170 kDa; ThermoFisher Scientific). Tris/glycine/SDS electrophoresis buffer (25 mM Tris–HCl, 192 mM glycine, 0.1% SDS, pH 8.3; Bio-Rad) was used for gel electrophoresis at 45 mA per gel for a minimum of 1 h.

Proteins were transferred from the SDS gels to methanol activated polyvinylidene difluoride (PVDF) membranes (GE Healthcare) at 120 V for 75 min in ice-cold transfer buffer (150 mM glycine, 20% [v/v] methanol and 25 mM Tris–HCl). After transfer, PVDF membranes were blocked with 5% (w/v) non-fat milk powder in PBS, 0.1% Tween-20 (PBST) for 30 min at RT. PVDF membranes were briefly washed twice with PBS and incubated with primary antibody diluted with PBST in milk or bovine serum albumin overnight at 4°C. The primary antibodies used were: Tau12 epitope residues 6–18 in tau, used at a dilution 1:4000, MAB2241 Merck; Tau5, epitope residues 218–225, dilution 1:1000, AHB0042 ThermoFisher Scientific; T46, epitope residues 404–441, dilution 1:1000, 13–6400 ThermoFisher Scientific; K9JA, epitope residues 243–441, dilution 1:4000, A0024 DAKO; T181, epitope phospho-T181, dilution1:1000, #12885 Cell Signalling; T231, epitope phospho-T231, dilution 1:1000, MAB5450 Merck; PHF-1, epitope phospho-S396/S404, dilution 1:500, P. Davies, Albert Einstein College of Medicine, U.S.A. The primary antibody was removed, the PVDF membrane washed three times in PBST for 10 min each, then incubated in horseradish peroxidase-conjugated goat anti-mouse (#31430) or goat anti-rabbit (#A16096) secondary antibodies (1:5000; 2% BSA/PBST; ThermoFisher Scientific) for 1 h at room temperature. Secondary antibody was removed, followed by two washes in PBST for 10 min each and a final wash in PBS prior to imaging using a G-Box (Syngene) with enhanced-chemiluminescent horse-radish peroxidase substrates according to the manufacturer's instructions (Pierce; ThermoFisher Scientific). PVDF membranes containing lysate samples were stripped and reprobed for β-actin as a loading control. PVDF membranes containing media samples were stained with Amido black staining solution (0.2% [w/v] Amido black, 40% [v/v] Methanol) to monitor protein loading.

For fluorescent immunoblotting, proteins were transferred to PVDF membranes and the membranes blocked as described above. The PVDF membranes were then incubated with the following primary antibody combinations overnight at 4°C: N-terminal tau antibody and T181 or K9JA and PHF-1. The primary antibody was removed, the PVDF membrane washed three times in PBST for 10 min each, then incubated in fluorescently-conjugated secondary antibodies (goat anti-mouse AzureSpectra 550 (1:5000; 2% BSA/PBST; Azure Biosystems; AC2159) and goat anti-rabbit AzureSpectra 650 (1:5000; 2% BSA/PBST; Azure Biosystems; AC2165) for 1 h in the dark at room temperature. The secondary antibodies were removed, the PVDF membrane washed twice in PBST for 10 min each and a final wash in PBS in the dark prior to imaging using the Azure C600 (Azure Biosystems). PVDF membranes were subsequently reprobed with β-actin as a loading control as described above. Densitometry of both chemiluminescent and fluorescent PVDF membranes was performed using the Genetools analysis software with manual background correction (Syngene).

### Dephosphorylation with lambda protein phosphatase

Clarified cell lysates or concentrated media (60 μg total protein) were incubated with 1000 units lambda protein phosphatase according to the manufacturer's instructions (New England Biolabs). The reaction was incubated for 3 h at 30°C, and stopped following the addition of SDS PAGE sample buffer and heating to 95°C for 5 min.

### Recombinant protein assays

Recombinant human GzmA (Biolegend) was activated in 100 mM Tris–HCl, pH 9.0 using 0.1 μg/ml Lysyl-Endopeptidase (Merck) at 37°C for 1 h. Activated GzmA was diluted to 20 ng in 50 mM Tris–HCl, pH 8.0 and incubated with 100 ng recombinant human tau_1-441_ (Sigma–Aldrich) for 1 h at 37°C in the absence or presence of 2 μM FUT-175. Reactions were stopped by the addition of SDS PAGE sample buffer, heated to 95°C for 5 min and immediately analysed by SDS–PAGE.

### Granzyme A ELISA

Pre-motor cortex tissue (200 mg/ml) from CBD cases 14/33 and 14/40, PSP cases 19/05, 17/33 and 13/06 and control cases 17/38, 18/03 and 18/05, and temporal cortex tissue (200 mg/ml) from AD cases 18/39, 19/13 and 19/04 and control cases 17/38, 18/03 and 18/05 (Table 2) were homogenised using an electrical homogeniser in homogenisation buffer (10 mM Tris–HCl, 0.8 M NaCl, 1 mM EGTA, 1 mM dithiothreitol, pH 7.5) with 4% (v/v) protease inhibitor cocktail and 10% (v/v) phosphatase inhibitor. The homogenate was centrifuged at 20 000 × ***g*** for 15 min at room temperature using a Beckman Optima MAX XP ultracentrifuge (TLA-110 rotor Beckman Coulter). The supernatant was removed as the total homogenate for determination of GzmA levels by ELISA. Samples were diluted 1:10 in reagent diluent before GzmA was measured using the GzmA ELISA kit (R&D Systems; DY2905) with the DuoSet Ancillary Reagent Kit 2 (R&D Systems; DY008) according to the manufacturer's instructions. Protein concentrations in each sample were determined using the BCA assay and the amount of GzmA in each sample reported as pg per mg of protein.

### Immunohistochemistry

Paraffin-embedded wax sections (6 μm thick) of human pre-motor cortex were used for immunohistochemistry. Staining was performed on the Leica Autostainer RX according to the manufacturer's instructions (Leica Biosystems). Bond polymer refine red detection kit was used for GzmA staining and bond polymer refine detection kit was used for NeuN staining (3, 3′-diaminobenzidine) followed by counterstaining with the blue nuclear stain, haematoxylin and eosin (H&E). After immunostaining, sections were immediately mounted using vectamount permanent mounting medium (Vector Laboratories) then dried prior to storage at room temperature. Sections were imaged using the Pannoramic 250 Flash Slide Scanner (3D Histech Ltd) at 40× magnification. Images were viewed and acquired in the CaseViewer 2.9 software (3D Histech Ltd) and representative images are shown.

### Mass spectrometric analysis of granzyme A-cleaved tau

Recombinant human tau_1–441_ (10 μg) was combined with 1 μg activated GzmA and incubated for 3 h at 37°C. The reaction was stopped and proteins separated by SDS–PAGE as described above. The SDS gel was stained with Coomassie Brilliant Blue (Bio-Rad) for 2 h at room temperature. The gel was then destained using 50% methanol, 40% acetic acid, 10% dH_2_O overnight with repeated changes in destain solution until the majority of the background staining had been removed. After destaining, the gel was repeatedly washed in dH_2_O and left overnight in fresh dH_2_O prior to analysis by liquid chromatography-mass spectrometry/mass spectrometry (LC–MS/MS). Bands were excised from the gel at 30 and 37 kDa then dehydrated using acetonitrile followed by vacuum centrifugation. Dried gel pieces were reduced with 10 mM dithiothreitol and alkylated with 55 mM iodoacetamide, then washed alternately with 25 mM ammonium bicarbonate followed by acetonitrile before being dried by vacuum centrifugation. Samples were digested with 12.5 ng/μl Asp-N overnight at 37°C in 25 mM ammonium bicarbonate. Digested samples were analysed by LC–MS/MS using an UltiMate® 3000 Rapid Separation LC (RSLC, Dionex Corporation, Sunnyvale, CA, U.S.A.) coupled to an Orbitrap Elite (ThermoFisher Scientific) mass spectrometer. Peptide mixtures were separated using a gradient from 92% (A; 0.1% formic acid in H_2_O) and 8% B (B; 0.1% formic acid in acetonitrile) to 33% B, in 44 min at 300 nl min^−1^, using a 75 mm × 250 μm internal diameter 1.7 μM BEH C18 analytical column (Waters). Peptides were selected for fragmentation automatically by data dependent analysis. Data produced were searched using Mascot (Matrix Science UK), against the Swissprot and Trembl databases with taxonomy of human selected and analysed against all enzyme digests. Data were validated using Scaffold version 4 (Proteome Software). Carbamidomethylation was set as a fixed post-translational modification of cysteine, while oxidised methionine and phosphorylation at serine, threonine and tyrosine residues were set as variable allowable modifications. Peptides of interest were analysed that corresponded to digestion by GzmA at one end and Asp-N at the other or a double digestion by GzmA. A minimum threshold for 50% was set for Scaffold peptide identification probability and b- and y-ion spectra were assessed visually (Supplementary Figure S2).

### Brain homogenisation

Sarkosyl-insoluble (SI) brain fractions were generated from brain samples using an established protocol [16,53]. For the mass spectrometric analysis of tau fragments, pre-motor cortex tissue (200 mg/ml) from PSP case 13/06 and temporal cortex tissue (200 mg/ml) from AD case 19/04 (Table 1) were used. For the seeding experiments, pre-motor cortex tissue (100 mg/ml) from control case 16/29, CBD case 10/32 and PSP case 12/31 (Table 1) were used. Samples were homogenised using an electrical homogeniser in homogenisation buffer (10 mM Tris–HCl, 0.8 M NaCl, 1 mM EGTA, 1 mM dithiothreitol, pH 7.5) with 4% (v/v) protease inhibitor cocktail and 10% (v/v) phosphatase inhibitor. The homogenate was centrifuged at 100 000 × ***g*** for 20 min at room temperature using a Beckman Optima MAX XP ultracentrifuge (TLA-110 rotor or MLA-130 rotor; Beckman Coulter). The supernatant was removed as the total soluble protein fraction. The pellet was sonicated using a handheld XL-2000 sonicator (Misonix) in 2 ml homogenisation buffer containing 1% Triton X-100 then incubated for 30 min at 37°C. After incubation, homogenates were centrifuged at 100 000 × ***g*** for 20 min at room temperature. The supernatant was removed and the pellet sonicated in 1.2 ml homogenisation buffer containing 1% sarkosyl and then incubated at 37°C for 30 min. After incubation, samples were centrifuged at 100 000 × ***g*** for 20 min at room temperature, the supernatant removed and the SI pellet sonicated either in 100 μl homogenisation buffer for immunoprecipitation of tau prior to mass spectrometry or in 240 μl 20 mM Tris–HCl, pH 7.5 which was stored at −80°C for the seeding experiments.

### Mass spectrometric analysis of tau fragments in human brain tissue

Protein G Dynabeads (Invitrogen) were washed in 1 ml PBS before incubation with 4 µg of each of the tau antibodies AT8 (tau epitope phospho-S202/T205, MN1020 Invitrogen) and tau5 in 500 µl PBS for 2 h at room temperature with rotation. Beads were recovered and washed in 1 ml PBS before incubation with 100 µl SI fraction prepared as described above for 2 h at 4°C with rotation. The beads were recovered, re-suspended in 100 µl PBS, transferred to a new tube, recovered again and washed three times in 1 ml PBS. The recovered beads were then resuspended in 50 µl 2X SDS PAGE sample buffer and heated at 95°C for 5 min. A 2 µl sample was taken to check successful immunoprecipitation of tau and the remaining 48 µl was minimally separated on SDS polyacrylamide gels and the protein bands corresponding to the identified fragments subjected to protease digestion with either Asp-N or Lys-C.

The digested samples were then analysed by nano-liquid chromatography-mass spectrometry. Peptide separation was performed on a Thermo RSLC system consisting of a NCP3200RS nano pump, WPS3000TPS autosampler and TCC3000RS column oven configured with buffer A as 0.1% formic acid in water and buffer B as 0.1% formic acid in acetonitrile. An injection volume of 2 μl was loaded into the end of a 5 μl loop and reversed flushed on to the analytical column (Waters nanoEase M/Z Peptide CSH C18 Column, 130 Å, 1.7 µm, 75 µm × 250 mm) kept at 35°C at a flow rate of 300 nl/min for 8 min with an initial pulse of 500 nl/min for 0.3 min to rapidly re-pressurise the column. The injection valve was set to load before a separation consisting of a multistage gradient of 1% B to 5% B over 2 min, 5% B to 21% B over 44 min, 21% B to 31% B over 7 min and 31% B to 65% B over 1 min before washing for 4 min at 65% B and dropping down to 2% B in 1 min. The complete method time was 75 min. The analytical column was connected to a Thermo Exploris 480 mass spectrometry system via a Thermo nanospray Flex Ion source via a 20 μm ID fused silica capillary. The capillary was connected to a stainless steel emitter with an outer diameter of 150 μm and an inner diameter of 30 μm (Thermo Scientific, ES542). The nanospray voltage was set at 2000V and the ion transfer tube temperature set to 275°C.

Data were acquired using a method that comprised of two MS experiments. The first experiment used data dependent acquisition using a fixed cycle time of 1.2 s, an expected peak width of 15 s and a default charge state of 2. Full MS data was acquired in positive mode over a scan range of 300 to 1750 Th, with a resolution of 120 000, a normalised automatic gain control target of 300% and a max fill time of 25 ms for a single microscan. Fragmentation data were obtained from signals with a charge state of +2 or +3 and an intensity over 5000 and they were dynamically excluded from further analysis for a period of 15 s after a single acquisition within a 10 ppm window. Fragmentation spectra were acquired with a resolution of 15 000 with a normalised collision energy of 30%, a normalised automatic gain control target of 300%, first mass of 110 Th and a max fill time of 25 ms for a single microscan. All data were collected in profile mode. The second experiment used a timed MS acquisition using a cycle time of 3 s. Data were collected with an isolation window of 2 Th, collision energy of 30% and a resolution of 30 000. Fragmentation data were acquired using the parameters detailed in Table 3.

**Table 3.** Parameters used for detection of tau peptides by mass spectrometry

Peptide	*m*/*z*	*z*	Retention time (min)	Window (min)
Tau-195–209	697.3207	2	25.75	5
Tau-210–224 4+	416.733	4	32	5
Tau-210–224 3+	555.3088	3	32	5
Tau-195–224 5+	608.5118	5	37.77	5
Tau-195–224 4+	760.6383	4	37.77	5
Tau-241–252	598.8264	2	47.78	5

Data analysis was performed using SkyLine software [54] version 22.2.0.527. The sequences of the peptides to be targeted were entered into the software in a FASTA format and the potential y, b, a and c ions with a +1 or +2 charge state were determined by SkyLine (peptides are non-tryptic and therefore a wide range of fragment types were considered). The data acquired from the peptide standards were loaded into Skyline and their retention times and five most intense fragments were determined (Table 4). Experimental data were assessed using this methodology and peptides were considered positively identified if co-eluting signals were seen for all five fragment ions with retention times within 0.2 min of the expected retention time.

**Table 4.** Parameters determined from analysis of standard peptide mix for detection of tau peptides by mass spectrometry using SkyLine software

Peptide	Sequence	Precursor mass and charge state	Retention time (min)	Fragment ions used for detection
Tau-195–209	SGYSSPGSPGTPGSR	697.3207 (2+)	25.8	912.4534 (y_10_), 671.3471 (y_7_), 416.2252 (y_4_), 999.4854 (y_11_), 1086.5174 (y_12_)
Tau-210–224	SRTPSLPTPPTREPK	555.3088 (3+)	32.0	824.4625 (y_7_), 412.7349 (y_7_^2+^), 727.4097 (y_6_), 925.5102 (y_8_), 1022.5629 (y_9_)
Tau-241–252	SRLQTAPVPMP	598.8264 (2+)	47.8	853.4990 (b_8_), 825.4941 (a_8_), 1053.5874 (a_10_), 1081.5823 (b_10_), 344.1639 (y_3_)
Tau-195–224	SGYSSPGSPGTPGSR SRTPSLPTPPTREPK	608.5116 (5+)	37.8	412.7349 (y_7_^2+^), 824.4625 (y_7_), 727.4097 (y_6_), 1022.5629 (y_9_), 511.7851 (y_9_^2+^)

### Seeding experiments

HEK cells were transiently transfected in Opti-MEM with empty vector or the cDNA encoding tau_1–441_, tau_1–194_, tau_195–441_, tau_1–209_ or tau_210–441_ for 24 h and then incubated for 6 h in Opti-MEM with or without 2.53 μl SI brain seed produced as described above. Media was then exchanged for DMEM and cells were further incubated for 48 h. Cells were then harvested in homogenisation buffer (10 mM Tris–HCl, 4 M NaCl, 1 mM EGTA, 1 mM DTT, pH 7.5) and subjected to SI fractionation as described above. The total soluble fraction was resuspended in SDS PAGE sample buffer and heated at 95°C for 5 min then separated by SDS–PAGE. The SI fraction was diluted in 70 μl 20 mM Tris–HCl buffer and either resuspended in SDS PAGE sample buffer, heated at 95°C for 5 min then separated by SDS–PAGE or stored at −80°C for further seeding experiments.

For the tau immunodepletion experiment, HEK cells were transiently transfected in Opti-MEM with the cDNA encoding tau_195–441_ for 24 h. After transfection, cells were treated with 10 μl (1:10) tau immunodepleted (using a combination of antibodies HT7, Tau5 and PHF-1) or IgG control-immunodepleted SI fractions from CBD case 10/32 (Table 1) diluted in 0.5 ml Opti-MEM for 6 h. Media was then exchanged to DMEM and cells were further incubated for 24 h. The cells were then harvested and prepared for SDS–PAGE.

For the propagation between cells experiment, the SI fraction from HEK expressing tau_195–441_ donor cells that had been seeded with either vehicle (Opti-MEM), control case 16/29, CBD case 10/32 or PSP case 12/31 (Table 1) were sonicated and 10 μl of the SI fraction was diluted in 0.5 ml OptiMEM. The diluted SI fraction from the donor cells was seeded into HEK acceptor cells expressing tau_195–441_ for 6 h. Media was then exchanged for DMEM and cells were further incubated for 48 h. The cells were then harvested and prepared for SDS–PAGE.

## Data Availability

The data supporting the findings reported in this paper are openly available from the University of Manchester FigShare repository at DOI 10.48420/26311240.

## Open Access

Open access for this article was enabled by the participation of University of Manchester in an all-inclusive *Read & Publish* agreement with Portland Press and the Biochemical Society under a transformative agreement with JISC.

## Abbreviations

AD: Alzheimer's disease
BCA: bicinchoninic acid
CBD: corticobasal degeneration
DMEM: Dulbecco's Modified Eagle's Medium
GzmA: granzyme A
GzmB: granzyme B
HEK: human embryonic kidney
MCI: mild cognitive impairment
MS: mass spectrometry
PAGE: polyacrylamide gel electrophoresis
PBS: phosphate-buffered saline
PBST: phosphate-buffered saline containing 0.1% Tween-20
PCR: polymerase chain reaction
PSP: progressive supranuclear palsy
PVDF: polyvinylidene difluoride
SDS: sodium dodecyl sulfate
SI: sarkosyl-insoluble
