# Supplementary material for: Proteolysis of tau by granzyme A in tauopathies generates fragments that are aggregation prone
Source: Biochem J. 2024 Sep 18;481(18):1255–74. doi: 10.1042/BCJ20240007 (PMC11555691; doi:10.1042/BCJ20240007)

Quinn et al. Proteolysis of tau by granzyme A in tauopathies generates fragments that are aggregation prone

Supplementary figures

Supplementary Figure 1. Identification of granzyme A-cleavage sites in tau.

Recombinant human $tau_{1\text{-}441}$ (10 µg) was incubated with activated recombinant GzmA (1 µg) for 3 h at 37°C. The incubation was then stopped by heating at 95°C for 5 min and the samples immediately separated by SDS-PAGE then stained with Coomassie Brilliant Blue. Bands in the molecular weight range 30-37 kDa were excised, subject to Asp-N digestion and analysed using LC-MS/MS.

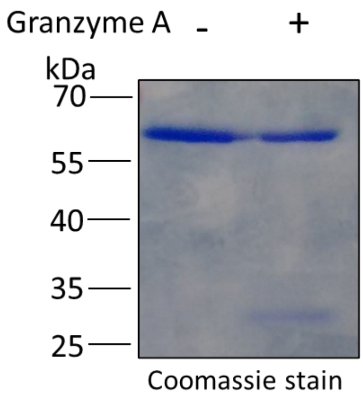

Supplementary Figure 2. Granzyme A cleaves tau at R194-S195, R209-S210 and K240-S241.

Detected MS/MS peptide spectra from excised bands from the Coomassie stained gel corresponding to GzmA-cleavage sites of $tau_{1\text{-}441}$ identified from the Scaffold4 software (Proteome Software; Portland, Oregon). B-ions are listed in red (top row), and y-ions are listed (bottom row) in blue. The detected peptide residues and their peptide identification probability are detailed above the peptide spectra. Potential GzmA cleavage sites of $tau_{1\text{-}441}$ are highlighted in bold.

195-209 $tau_{1\text{-}441}$ peptide (**R194-S195** and **R209-S210**; 94%)

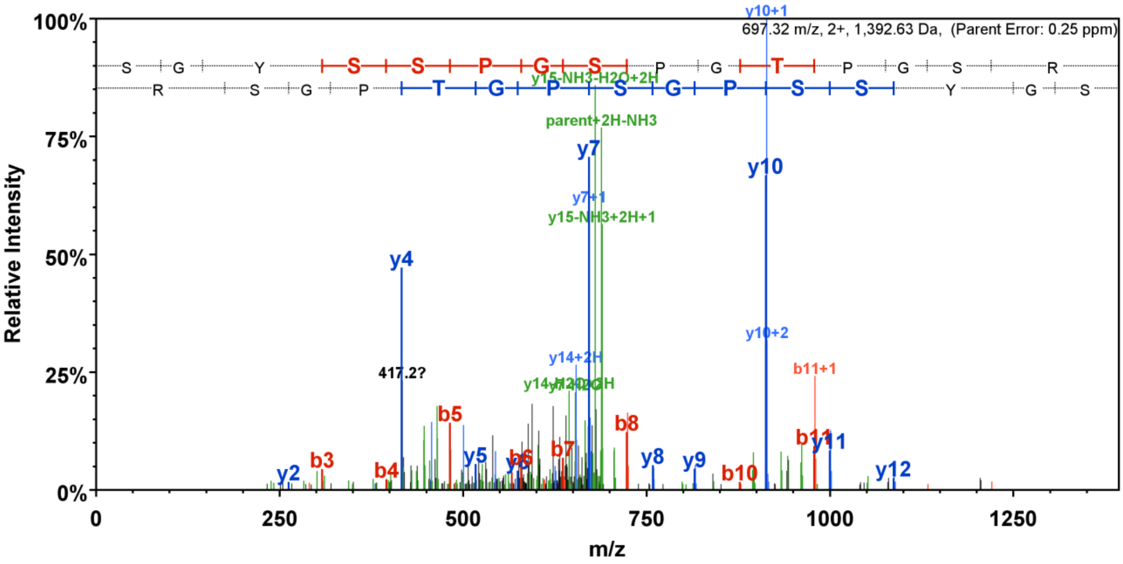

193-209 tau1-441 peptide (G192-D193 and **R209-S210**; 100%)

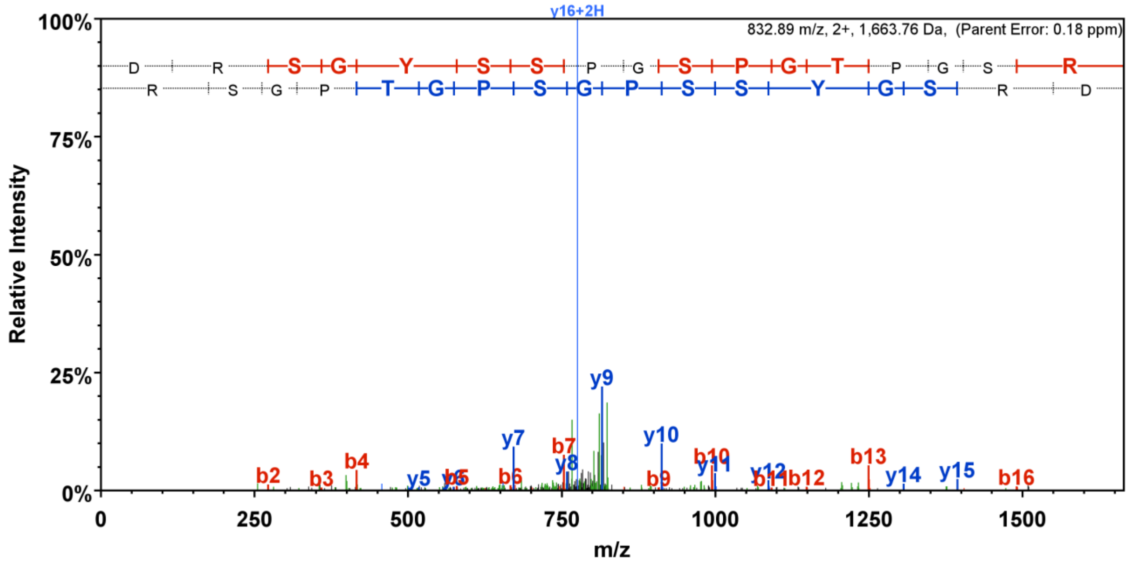

241-251 tau1-441 peptide (**K240-S241** and P251-D252; 65%)

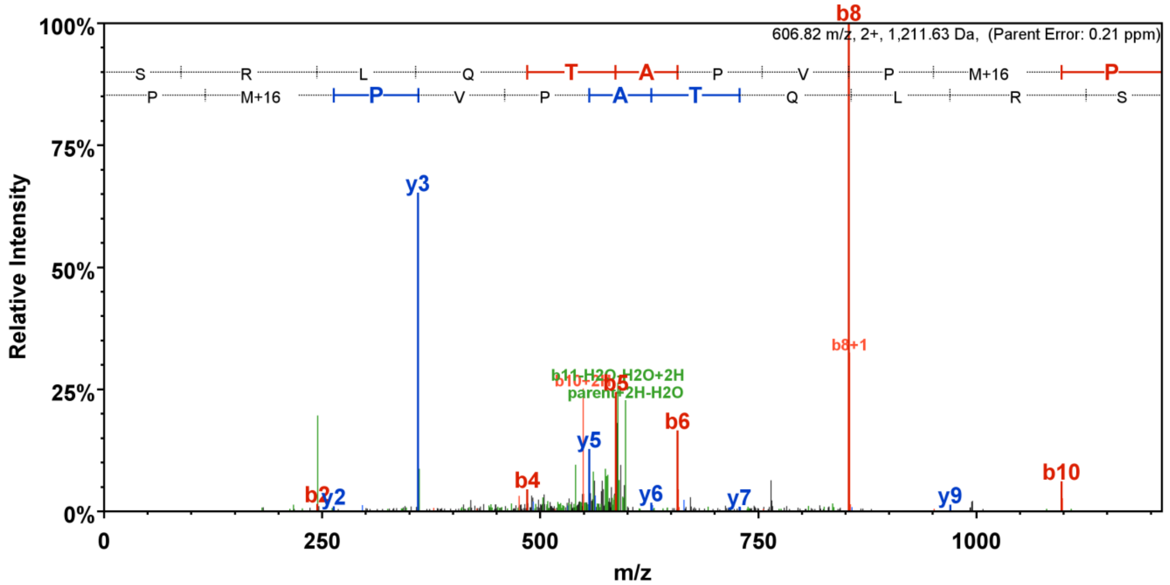

Supplementary Figure 3. Granzyme A cleaves tau to generate C-terminal fragments.

HEK293 cells were transfected with the cDNA encoding full-length tau and with the cDNA encoding GzmA and incubated in the absence or presence of 50 µM FUT-175 for 24 h. Cells and conditioned media were harvested, lysates prepared and conditioned media concentrated for analysis. (A) Lysates were immunoblotted with the C-terminal tau antibody K9JA (top) and actin (bottom). (B) Conditioned media was immunoblotted with the C-terminal tau antibody K9JA. Red arrows indicate the position of full-length tau, and green arrows the position of C-terminal fragments.

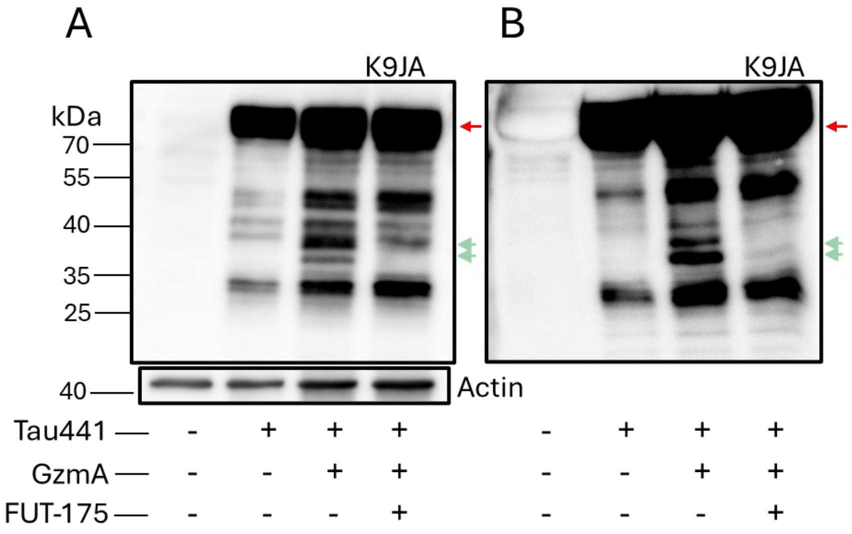

Supplementary Figure 4. Skyline chromatograms for Peptide Tau-210-224.

The five most abundant fragment ions observed, as listed in Supplementary Table 2. Both 3+ (upper) and 4+ (lower) charge states were detected. Left image: The peptide in the standard mix. Middle image: The peptide in the AD brain sample digested with LysC. Right image: The peptide in the PSP brain sample digested with LysC.

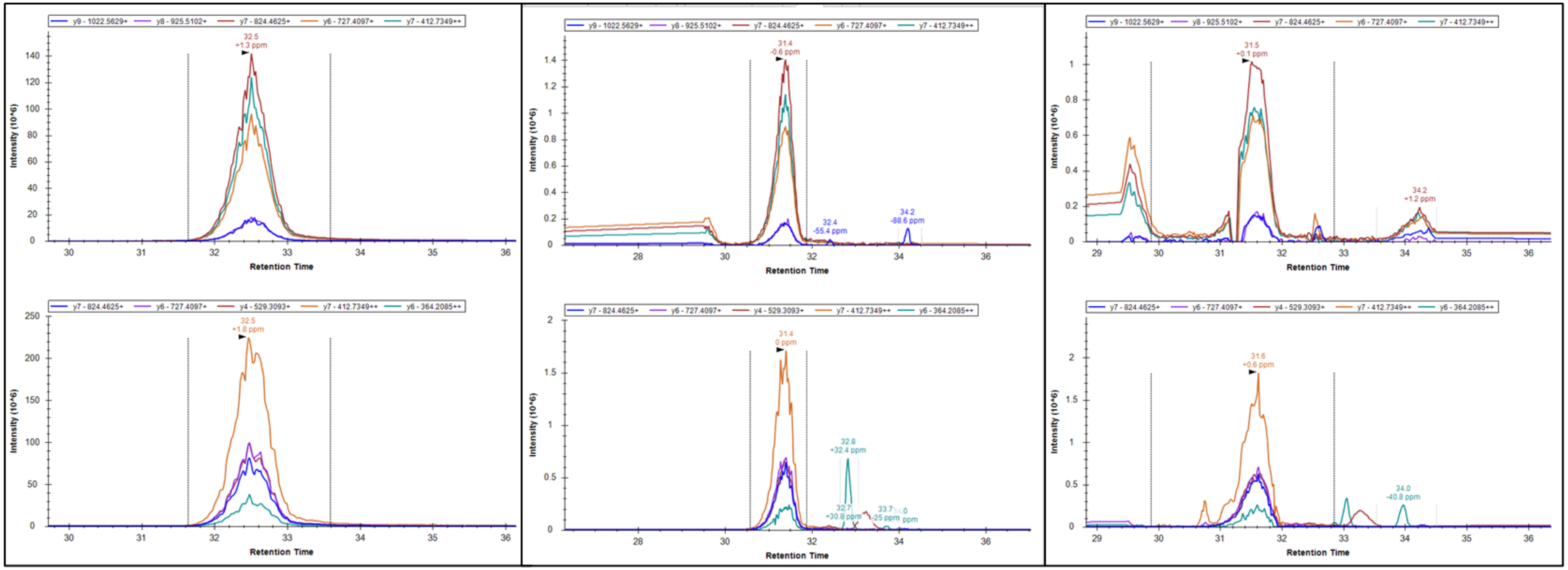

Supplementary Figure 5. Skyline chromatograms for Peptide Tau-241-252.

The five most abundant fragment ions observed, as listed in Supplementary Table 2. Charge state 2+ was detected. Left image: The peptide in the standard mix. Middle image: The peptide in the AD brain sample digested with AspN. Right image: The peptide in the PSP brain sample digested with AspN.

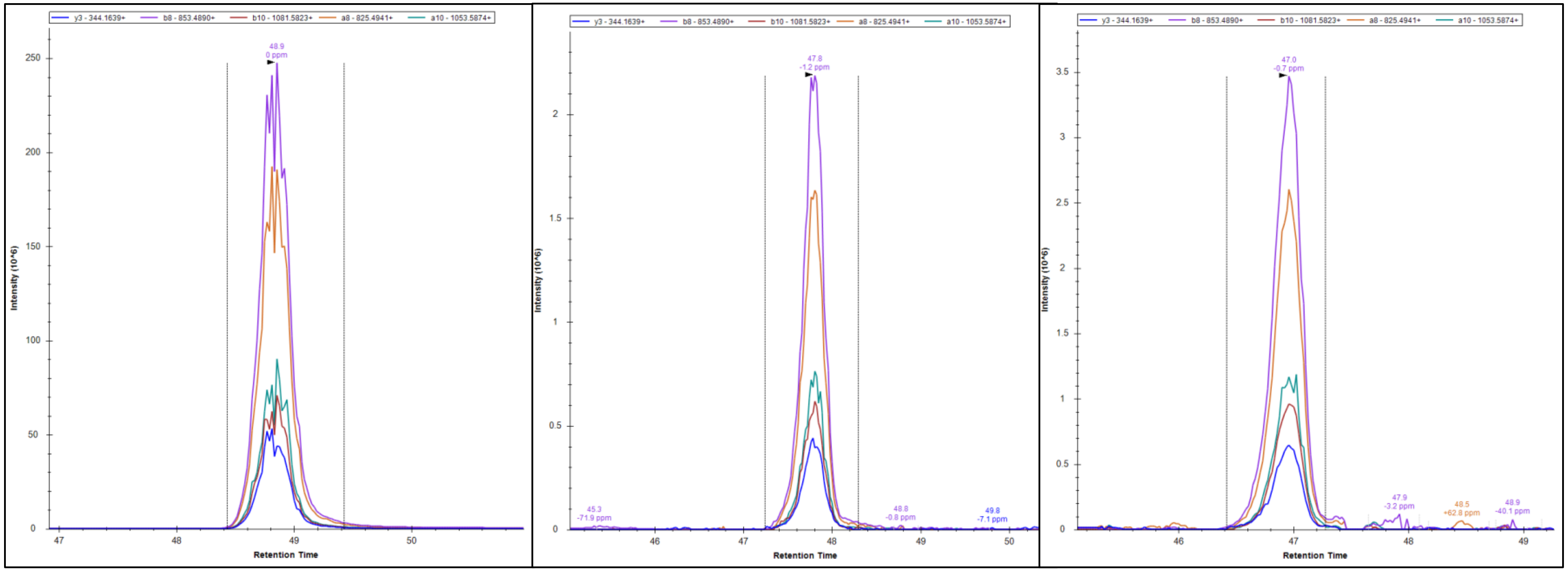

Supplement: Supplementary Material [file BCJ-481-1255-s1.pdf]
